# Proteotoxic Stress Bioreporter Enables Mechanism-Informed Antibiotic Discovery

**DOI:** 10.1021/acs.jnatprod.6c00112

**Published:** 2026-05-15

**Authors:** Julian Schubert, Christian Geibel, Anne Berscheid, Katharina W. Wex, Giovanni Andrea Vitale, Chambers C. Hughes, Daniel Petras, Heike Brötz-Oesterhelt

**Affiliations:** † Department of Microbial Bioactive Compounds, Interfaculty Institute of Microbiology and Infection Medicine (IMIT), 9188 University of Tuebingen, 72076 Tuebingen, Germany; ‡ Cluster of Excellence EXC 2124: Controlling Microbes to Fight Infection (CMFI), University of Tuebingen, 72076 Tuebingen, Germany; § German Center for Infection Research (DZIF), Partner Site Tuebingen, 72076 Tuebingen, Germany; ∥ Department of Biochemistry, University of California Riverside, Riverside, California 92507, United States

## Abstract

Antibiotic resistance poses a major global health challenge, which is intensified by the decline in antibiotic discovery efforts. This highlights the urgent need for innovative antibacterial agents. Natural products remain a key source of antibiotics, yet early mechanistic insights are often lacking. Classical whole-cell screening identifies growth inhibition but provides no mechanistic information. Bioreporter strains combine bioactivity detection with preliminary mechanistic profiling; however, they remain unavailable for several promising antibiotic target areas. We developed a sensitive bioreporter that signals the induction of the heat-shock Clp-ATPase gene *clpE* in *Bacillus subtilis*. ClpE serves as a specific biomarker for proteotoxic stress, resulting from the accumulation of damaged or misfolded proteins. The self-sustained luminescence of the P_*clpE*_-lux bioreporter enables monitoring of proteotoxic stress in both solid and liquid assay formats and supports high-throughput screening. Consolidated through extensive validation with reference antibiotics, the bioreporter also identified antibacterial agents that were not previously associated with proteotoxic stress. Coupling the bioreporter with a microfractionation-based metabolomics workflow accelerated the identification of hydrophilic antibacterial metabolites from complex natural product extracts, thereby uncovering an extensive group of streptothricin derivatives, including putatively novel analogues. This study demonstrates that the P_*clpE*_-lux bioreporter is a versatile tool for mechanism-informed antibiotic discovery, enhancing the detection sensitivity and dereplication efficiency.

## Graphical abstract

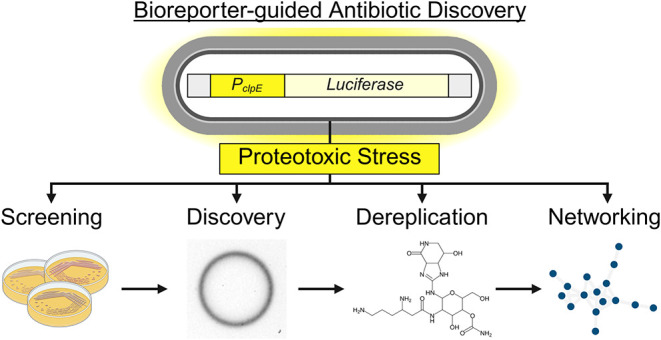

The global threat of antibiotic-resistant pathogens necessitates the development of new antibacterial agents, yet the discovery of novel antibiotic classes remains rare, and approval rates continue to decline.^1,2^ Sustained clinical efficacy requires a robust discovery and development pipeline capable of delivering antibiotic candidates that meet essential criteria, including novelty in chemical classes and mechanisms of action (MoAs) and the absence of cross-resistance.^3,4^ In past decades, high-throughput screening campaigns with synthetic libraries and purified protein targets conducted by the pharmaceutical industry have yielded very few new antibacterial leads. Severe limitations included a lack of whole-cell activity due to limited cell penetration, nonspecific activity, and pharmacokinetic liabilities.^5,6^ To revitalize antibiotic discovery, contemporary drug discovery programs employ a broad range of complementary strategies. Assessment of microbial diversity in underexplored environments expands natural product discovery,^7^ while bioinformatic genome mining provides access to novel, silent, or cryptic biosynthetic pathways.^8^ The integration of multiomics, machine learning, and artificial intelligence further accelerates compound discovery, drug design, and target prediction.^9,10^ In parallel, ongoing advances in mass spectrometry-based dereplication strategies have improved efficiency in identifying antibacterial agents. Metabolic engineering increases secondary metabolite yields, supports heterologous expression of biosynthetic gene clusters, and enables cultivation of challenging producer strains.^11^ Semisynthetic optimization of natural products improves their potency, pharmacokinetics, or resistance profiles, while synthetic chemistry enables the rational design and total synthesis of new molecular scaffolds.^12^ Combination therapies exploit synergistic interactions,^13^ and drug repurposing uncovers additional antibacterial applications for drugs approved for other indications.^14,15^

Classical growth inhibition assays remain the primary approach in antibacterial natural product screening workflows due to their simplicity and proven effectiveness but are limited by labor-intensive sample preparation, modest throughput, low specificity, and frequent rediscovery of known compounds.^16,17^ Whole-cell assays evaluate compound efficacy in the physiological context of the bacterial cell, thereby accounting for key barriers such as cell envelope penetration and intracellular retention.^18,19^ A promising development in whole-cell screening involves the use of bioreporters, engineered bacterial strains that generate measurable reporter signals in response to antibiotic-induced stress. Antibiotic interference with biosynthetic processes triggers the upregulation of specific genes as a compensatory mechanism. Such genes can be repurposed to probe the stress-bearing pathway by fusing their promoters to reporter genes such as β-galactosidases or luciferases. Consequently, bioreporters provide a direct readout of antibacterial activity while simultaneously indicating the type of cellular stress and the affected target pathway. Additionally, bioreporters detect physiological damage at compound concentrations well below growth-inhibitory levels,^20^ enabling the identification of promising candidates that are overlooked by traditional inhibition assays.^21^

The Gram-positive model organism *Bacillus subtilis* was chosen for the development of antibiotic stress bioreporters due to its rapid growth, natural competence, broad antibiotic susceptibility, and well-characterized physiological stress responses to antibiotic exposure.^22–24^ Genome-wide analyses of antibiotic-induced mRNA expression profiles revealed modular gene expression patterns linked to distinct antibiotic mechanisms and identified promoters responsive to specific kinds of antibiotic-induced stress.^23,25^ Based on these insights, Urban et al. engineered a panel of firefly luciferase-based whole-cell bioreporters that detect cell envelope damage and inhibition of RNA, DNA, fatty acid, and protein syntheses.^22^ However, the protein synthesis bioreporter *bmrC* (formerly *yheI*) detects only ribosomal stalling (translation arrest) and does not sense proteotoxic stress caused by mistranslation, protein misfolding, or other forms of protein damage.^20–22^ Consequently, many natural products that induce proteotoxic stress, including clinically relevant aminoglycosides, remain undetected by the existing bioreporter panel. To address this gap in bioreporter coverage, we revisited the transcriptome data. We selected the heat-shock Clp-ATPase gene *clpE* as a candidate biomarker for proteotoxic stress in *B. subtilis* because it showed strong induction in response to aminoglycosides and *N*-ethylmaleimide.^23^ Under nonstress conditions, *clpE* is tightly repressed by the global stress regulator CtsR. Upon heat shock or proteotoxic stress, ClpE is rapidly upregulated and, in conjunction with the proteolytic core ClpP, forms the ClpEP protease complex. This protease contributes to protein quality control and the restoration of cellular proteostasis by degrading heat-denatured or otherwise damaged proteins.^26–30^ Given its functional role and its induction by agents that cause protein damage or misfolding, we evaluated *clpE* as a specific biomarker of proteotoxic stress.

Here, we report the construction and validation of a novel *B. subtilis* bioreporter based on the *clpE* gene, which is selectively induced by proteotoxic stress arising from the accumulation of damaged or misfolded proteins. By fusing the *clpE* promoter region (P_*clpE*_) to a bacterial luciferase operon, we generated the P_*clpE*_-lux bioreporter construct and established two complementary whole-cell assay formats in solid and liquid media. These assays enable convenient high-throughput screening of pure compounds, crude extracts, and natural producer strains without extensive sample preparation. Application of the P_*clpE*_-lux bioreporter to a library of diverse natural products identified several antibacterial agents that had not previously been linked to proteotoxic stress. Integration of the P_*clpE*_-lux bioreporter into a high-resolution microfractionation-based dereplication workflow^20^ enabled simultaneous dereplication and mechanistic characterization of antibacterial metabolites, including those present in highly hydrophilic fractions. This approach uncovered a substantial network of diverse streptothricin derivatives among 53 bacterial isolates from the Tuebingen collection of actinomycete producer strains, including analogues with putatively novel acetylation patterns.

## Results and Discussion

### Validation of the P_*clpE*_-lux Bioreporter Induction Profile

The P_*clpE*_-lux bioreporter was designed to expand the detection capabilities of our bioreporter panel by selectively identifying antibiotics that induce proteotoxic stress. For this purpose, the antibiotic-inducible P_*clpE*_ promoter was cloned upstream of a self-sustained bacterial luciferase operon (Figure 1A). Following chromosomal integration into a sporulation-deficient *B. subtilis* strain,^31^ induction specificity was validated against a panel of 99 antibacterial reference compounds with diverse, well-characterized MoAs (Table S4). The bioreporter enabled the selective detection of proteotoxic stress in liquid and solid assay formats. In the liquid format, luminescence signals were continuously monitored across a serial dilution of each reference compound through several generations of exponential growth. The agar-based assay format enabled higher throughput by detecting luminescence signals at a single time point and utilizing concentration gradients generated by compound diffusion in the agar. In both formats, the bioreporter showed robust induction in response to all tested compounds known to trigger proteotoxic stress (Figures 1B,1C and S1). P_*clpE*_-lux inducers include antibiotics with ribosome-dependent MoAs, such as aminoglycosides, which trigger translational misreading by compromising codon–anticodon recognition,^32^ and puromycin, which causes premature peptide chain termination through covalent incorporation into nascent polypeptides.^33^ Additionally, compounds with protein-damaging mechanisms independent of ribosomal interaction induce the bioreporter, including acyldepsipeptide antibiotics (ADEPs), *N-*ethylmaleimide (NEM), and tetramethylazodicarboxamide (TMAD). ADEPs deregulate the Clp protease, compromising protein homeostasis and regulatory proteolysis as well as promoting nonspecific degradation of unstructured and nascent polypeptides.^34,35^ Thiol-reactive electrophilic compounds, such as NEM and TMAD, covalently modify or oxidize cysteine residues and induce disulfide cross-linking.^36–39^ Compared with other reference compounds, NEM produced an unusually early bioreporter signal in the liquid assay. Also on agar, the luminescence signal was already detectable after 90 min but not at 180 min, indicating a rapid but short-lived proteotoxic stress response (Figure 1C).

**Figure 1:**
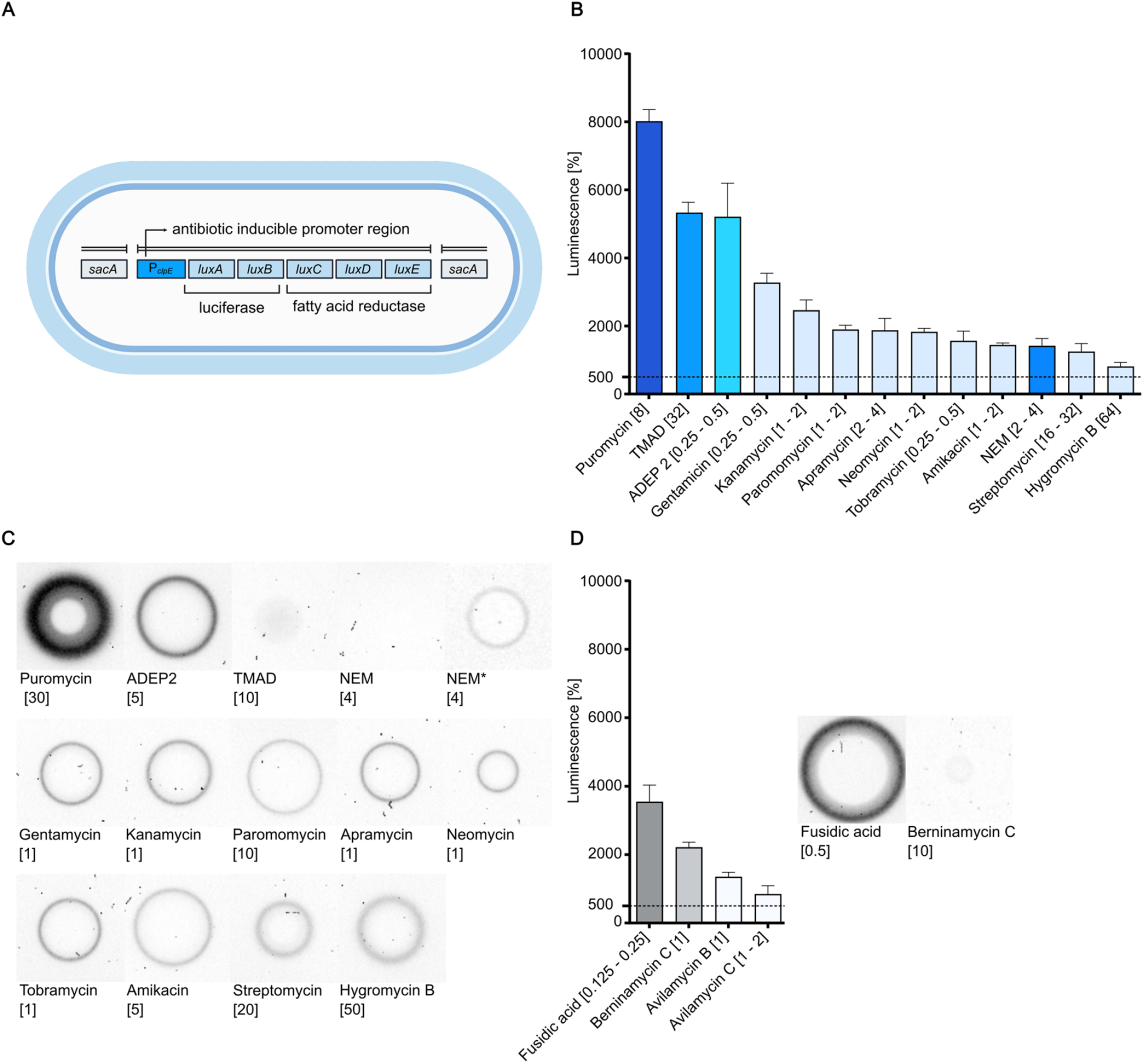
P_*clpE*_-lux bioreporter construction and validation. (A) Schematic of the P_*clpE*_-lux bioreporter integrated into the *B. subtilis* 1S34 *sacA* locus. P_*clpE*_ promoter insertion upstream of the *Photorhabdus luminescens* *luxABCDE* operon.^45^ (B) P_*clpE*_-lux induction by reference compounds in the liquid assay format, color-coded according to MoA: puromycin (dark blue), TMAD and NEM (blue), ADEP 2 (turquoise), and aminoglycosides (light blue). Compounds were tested in 2-fold serial dilutions, from below to above their minimal inhibitory concentration (MIC). Luminescence and OD_600_ (cell mass) were monitored for 180 min, and induction was normalized to the baseline luminescence of the untreated control (100%) and OD_600_. The induction threshold was defined as ≥500% luminescence within 90 min of antibiotic exposure (black dotted line). Extended incubation times occasionally resulted in nonspecific activation. Bars represent maximal normalized luminescence (mean ± standard deviation (SD)), and values in brackets indicate the compound concentration range at which those inductions occurred in independent biological replicates. (C) P_*clpE*_-lux induction by reference compounds in the agar-based assay format, with amounts [μg] spotted onto the bioreporter lawn. Luminescence imaging after 180 min showed bioreporter induction as luminescent halos at the margins of growth inhibition zones, with size and intensity depending on the amount of compound applied and its diffusion characteristics through the agar matrix. Transient NEM induction at 90 min (asterisk). (D) P_*clpE*_-lux induction by reference compounds not previously associated with proteotoxic stress: fusidic acid (dark gray), berninamycin C (gray), and avilamycin B and C (light gray) in liquid; fusidic acid and berninamycin C on agar. Bars and values in brackets are as in panel (B).

None of the reference compounds targeting DNA replication, RNA transcription, cell wall synthesis, or fatty acid biosynthesis induced the P_*clpE*_-lux bioreporter, nor did most agents that were classified as translation stallers. Such negative responses were characterized by luminescence signals well below the defined induction threshold in the liquid assay and no detectable luminescence signals on agar plates (Table S4, Figures S2–S5). Interestingly, induction of the P_*clpE*_-lux bioreporter was also observed for four compounds, among them two from the same class, whose primary MoAs were reported to be translation inhibition via ribosomal stalling rather than induction of proteotoxic stress. Fusidic acid, berninamycin C, and avilamycin B and C induced the bioreporter in the liquid assay, and fusidic acid and berninamycin C produced inductions on agar (Figures 1D, S2, and S5A,B). Fusidic acid targets elongation factor G (EF-G) bound to the ribosome, stabilizing EF-G in a conformation that prevents its dissociation after the guanosine triphosphate (GTP) hydrolysis. This inhibition stalls translocation and blocks subsequent ribosomal subunit recycling by locking EF-G in a posttranslocation state,^40,41^ leading to induction of the translation arrest biomarker *bmrC*.^21,22^ The P_*clpE*_-lux induction that we observed here aligns with increased *clpE* expression in previously reported transcriptomic data,^23^ thereby confirming *clpE* upregulation in response to fusidic acid and suggesting proteotoxic stress as an additional component of its MoA. For fusidic acid, it is reported that inhibition of ribosome recycling leads to the accumulation of ribosomes arrested in the posttermination ribosomal complex,^40^ which may explain the observed P_*clpE*_-lux induction, as similar secondary effects are also characteristic of aminoglycoside-induced stress.^42^ The cyclic thiopeptide berninamycin C also induced *bmrC*,^21^ in agreement with its established MoA of ribosome stalling through inhibition of amino acid incorporation into nascent polypeptides,^43^ but the induction of proteotoxic stress noted here has not been reported before. Berninamycin C contains dehydroalanine, a thiol-reactive moiety that could explain the occurrence of proteotoxic stress. However, other dehydroalanine-containing thiopeptides, such as thiostrepton and sulfomycin I, did not activate the P_*clpE*_-lux bioreporter, distinguishing berninamycin C as a notable exception in this class (Table S4). The orthosomycin antibiotics avilamycin B and C are known to disrupt both translation initiation and elongation by inhibiting formyl-methionyl-tRNA recruitment to the initiation complex and aminoacyl-tRNA positioning, respectively.^44^ P_*clpE*_-lux induction by fusidic acid, berninamycin C, and the avilamycins was consistently well above the induction threshold, with the signal strength comparable to those of agents with established proteotoxic MoAs. This argues against nonspecific bioreporter activation and encourages the hypothesis that damaged proteins may accumulate as an additional aspect of their MoA. The role of the bioreporter as an easy-to-handle entry assay is to point to such putative effects, which must be validated in follow-up studies by using orthogonal assay technologies.

The previously characterized translation arrest biomarker *bmrC* ^20–22^ and the new *clpE* biomarker exhibit complementary activation patterns. To date, only fusidic acid, berninamycin C, and hygromycin B^21^ have been documented to induce both bioreporters. Avilamycins may be another example, but they have not yet been tested on *bmrC*. Hygromycin B is an atypical aminoglycoside that primarily stalls the ribosome rather than promotes translational misreading,^42^ consistent with its low induction of P_*clpE*_-lux. These results underscore that the two bioreporters respond to distinct cellular stress pathways and can be used to distinguish proteotoxic stress from that of translation arrest. Taken together, our findings indicate that the P_*clpE*_-lux bioreporter functions as a physiological readout of proteotoxic stress by reporting the intracellular accumulation of damaged or misfolded proteins. Bioreporter activation can occur via ribosome-dependent or -independent mechanisms, and proteotoxic effects can be the immediate consequence of engagement of the primary target, downstream effects following target inhibition, or secondary effects that perturb protein homeostasis.

### Natural Product Library Screening for Proteotoxic Stress Inducers

A curated collection of 340 diverse natural products from the Tuebingen subset of the German Center for Infection Research (DZIF) natural product library was screened with the P_*clpE*_-lux bioreporter to identify additional antibacterial agents that induce proteotoxic stress. This subset contained natural products from actinomycetes (∼88%), fungi (∼6%), and a few agents from myxobacteria, plants, and other bacterial sources, including *Erwinia*, *Pseudomonas*, and *Staphylococcus*. A small number of synthetic compounds (2%) was also included. In total, 22 compounds elicited reproducible inductions, including derivatives of compound families (Figure 2). Consistent with bioreporter validation, the established inducers puromycin and berninamycin D were successfully rediscovered. Bioreporter induction was also observed for cervimycin K, in line with published omics data showing effective derepression of heat-shock response genes and transcriptional profiles resembling gentamicin-induced stress.^46^ Comparable to activation patterns observed for other thiol-reactive compounds such as NEM and TMAD, the thiol-reactive enzyme inhibitor naphthomycin A^47^ elicited robust induction, whereas its structural analogue naphthomycin B produced a weaker response.

**Figure 2:**
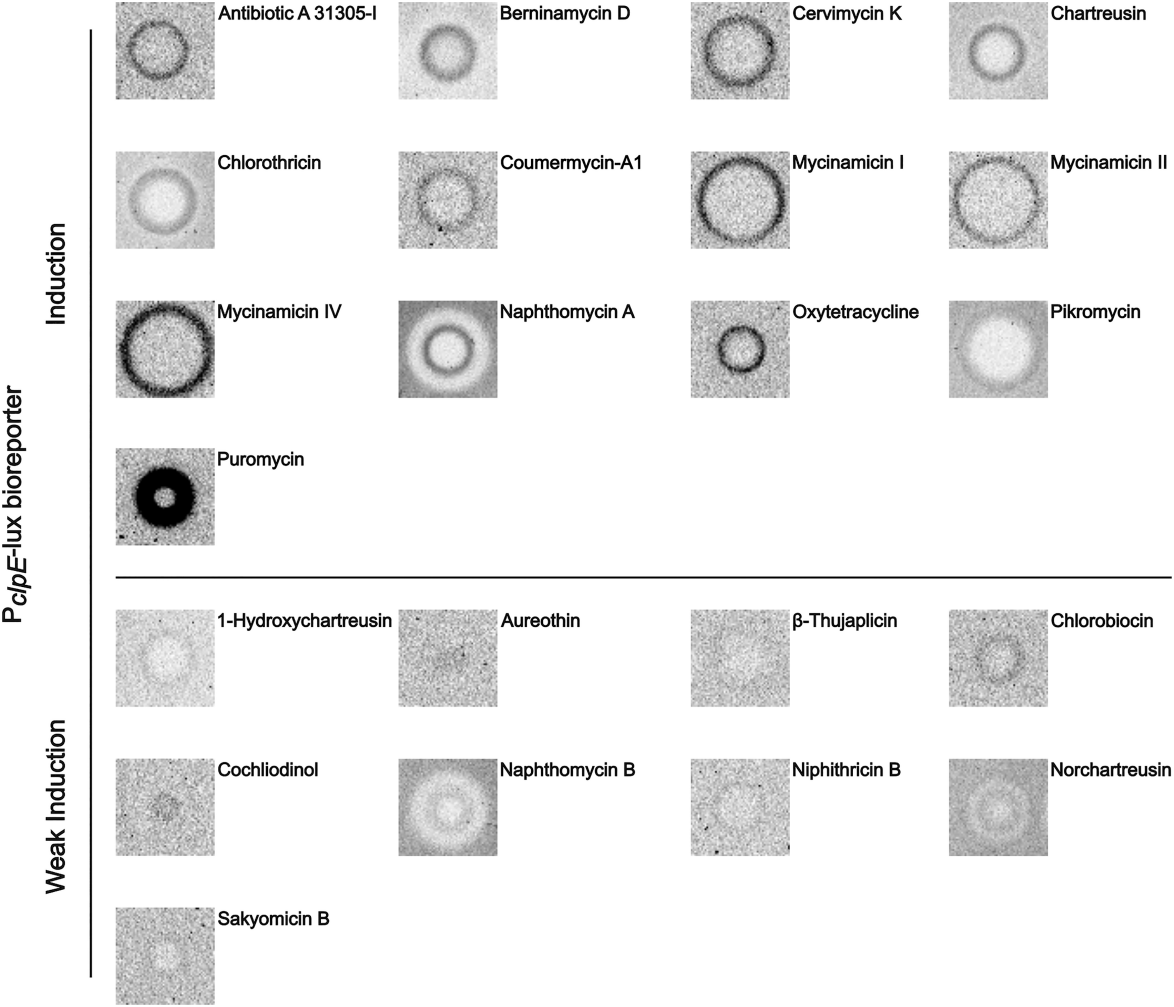
Proteotoxic stress induction by diverse natural products. Among the Tuebingen subset of the DZIF library (complete list of the 340 tested compounds provided in Supporting Information File 1), 22 natural products showed reproducible P_*clpE*_-lux induction, among them 13 strong and 9 weak inducers. Compounds were directly spotted onto the bioreporter lawn. Luminescence was imaged after 180 min and normalized to the untreated background. Representative uncropped screening plates are provided in Figure S6.

Several compounds whose primary MoAs are not typically associated with proteotoxic stress also induced the P_*clpE*_-lux bioreporter. This included the glycoside antibiotic chartreusin,^48,49^ whose pleiotropic MoA has previously been noted to induce the three bioreporters that signal DNA and RNA stress, as well as translation stalling.^21^ Given that antimicrobial compounds generally activate only a single bioreporter corresponding to their primary MoA, or a second due to overlapping or secondary activities,^21,22^ the induction of four distinct bioreporters is an exception, establishing chartreusin as the compound with the broadest bioreporter induction profile that we have observed to date. Structurally related derivatives showed reduced induction intensities, including 1-hydroxychartreusin and norchartreusin. Again, known translation stallers were among the inducing agents, including oxytetracycline, which inhibits aminoacyl-tRNA binding,^50^ and the macrolides mycinamicin I, II, and IV, and pikromycin, which interfere with nascent polypeptide progression through the exit tunnel.^51,52^ For mycinamicin and pikromycin, the P_*clpE*_-lux induction could potentially result from thiol-reactive electrophilic sites that both agents contain. Also, the spirotetronate polyketide chlorothricin, a known pyruvate carboxylase inhibitor,^53^ produced a strong response, suggesting protein damage as an additional mechanism. Among DNA gyrase-inhibiting aminocoumarin antibiotics,^54^ coumermycin-A1 elicited a moderate and chlorobiocin a weak response. In contrast, novobiocin, which was included in the validation set (Table S4, Figure S4), did not induce the bioreporter, suggesting that aminocoumarins differ in their secondary effects. For β-thujaplicin,^55^ we observed a weak induction, adding to the pleiotropic effects already reported for this compound. Additional P_*clpE*_-lux responses were triggered by compounds with largely uncharacterized MoAs, including the strong inducer antibiotic A31505-I, and the weak inducers aureothin, cochliodinol, niphithricin B, and sakyomicin B.

Overall, the rediscovery of established proteotoxic stress inducers corroborated the specificity of the P_*clpE*_-lux bioreporter. For antibiotic classes not typically associated with proteotoxic stress, P_*clpE*_-lux induction points to compound-specific secondary effects beyond their established MoAs. For antibacterial agents with elusive MoAs, the bioreporter helps to build hypotheses for subsequent MoA studies by complementary methods. The superior sensitivity of the bioreporter enabled the detection of weak compound activities that would have been overlooked in classical growth inhibition assays. Because of their relatively low induction levels, such agents are considered putative proteotoxic stress inducers and warrant further confirmation. It is also noteworthy that proteotoxic stress can be caused by a defined protein–target interaction as well as by a more general thiol-reactive (covalent) property of the compound, similar to maleimide-type reactivity.

### P_*clpE*_-lux Bioreporter-Guided Compound Dereplication and Discovery Pipeline

Following comprehensive validation, the potential of the bioreporter for antibiotic discovery was evaluated by screening natural producer strains from the Tuebingen actinomycetes collection. In a prior bioreporter-guided screening campaign, 137 isolates from the collection exhibited antibacterial activity against *B. subtilis* but did not induce the previous bioreporter panel (signaling DNA, RNA, cell wall, and translation stalling stress).^21^ We then applied the P_*clpE*_-lux bioreporter to these strains. In the screening phase, strains were cultivated on different solid media to stimulate secondary metabolite production. Agar plugs from well-grown cultures were extracted and embedded in bioreporter-containing agar, thereby circumventing the need for compound isolation (Figure 3A). ISP3 medium supported the most robust production of proteotoxic stress-inducing metabolites among this collection, yielding consistent bioreporter inductions in 53 isolates. Biomass from these strains was subjected to ethyl acetate–water partitioning, and both the aqueous and organic fractions were reassessed for bioreporter induction. Bioreporter signals were mainly observed in the aqueous extracts. For dereplication, we used a novel bioreporter-guided compound dereplication and discovery pipeline. Details of the development, validation, and application of this compound-resolved, bioactivity-based metabolomics workflow were recently reported.^20^ Here, we demonstrate its application for the dereplication of the bioactive compound contained in the aqueous extract of producer strain Tue2, using nontargeted high-performance liquid chromatography-tandem mass spectrometry (HPLC-MS/MS) coupled with high-frequency microfractionation onto a microfluidic paper-based analytical device (μPAD). Due to the high polarity of the bioactive metabolites, chromatographic separation was performed on a Hypercarb column, as conventional reversed-phase (RP) failed to retain the bioreporter-active agents. For extract separation, the gradient was held for 2 min at a low initial concentration of the organic modifier (2%) to enhance retention of polar analytes through the polar retention effect on graphite, a characteristic feature of this stationary phase.^56^ Microfractionation was performed by splitting the column eluent using a T-piece, enabling μPAD spotting at 1 Hz at a flow rate of 1 mL/min (∼15 μL per spot, splitting ratio 9:1 microspotter/MS), while simultaneously acquiring corresponding MS/MS data.

**Figure 3:**
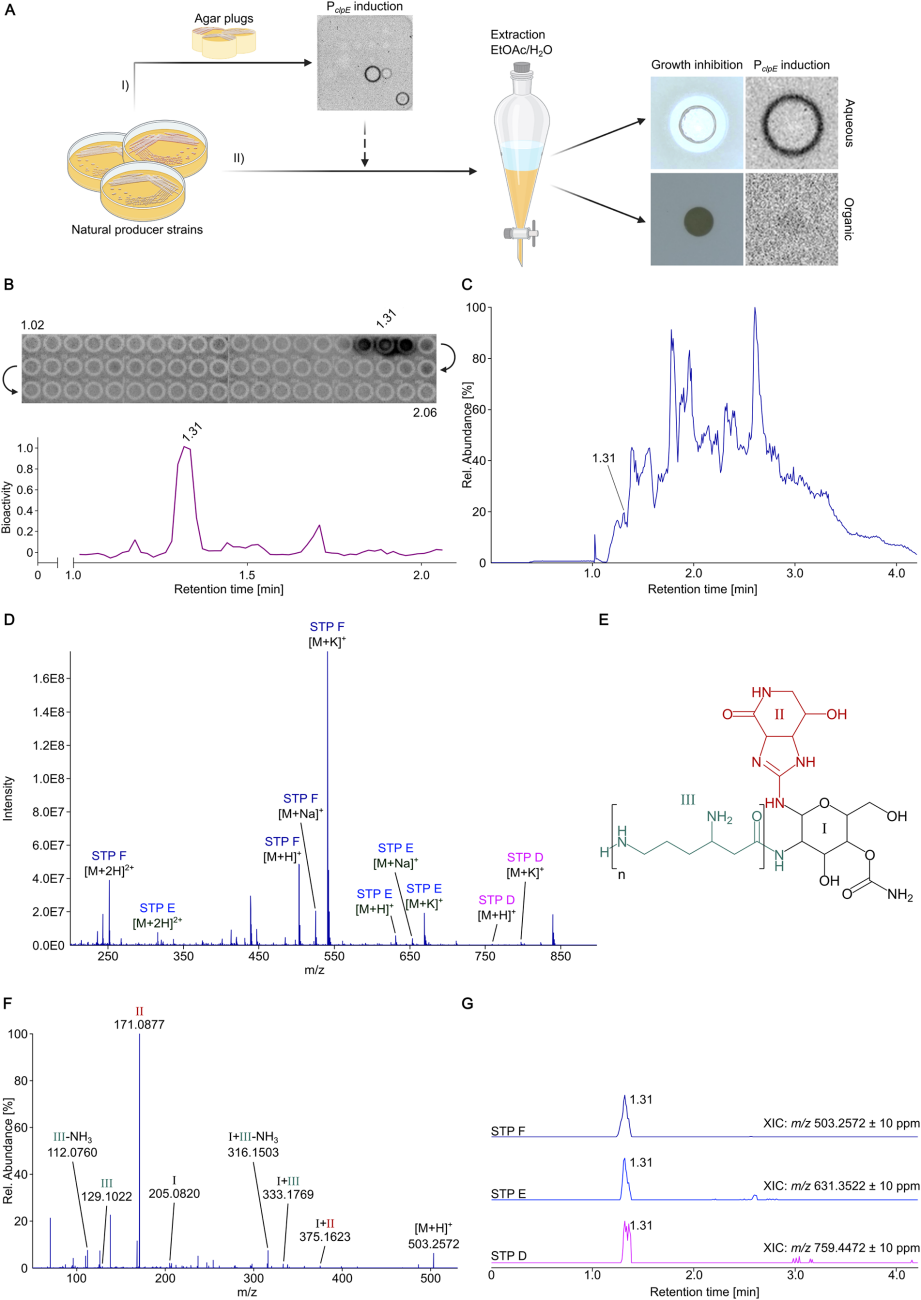
Bioreporter-guided compound dereplication and discovery pipeline. Workflow illustrating the identification of bioactive metabolites from the aqueous extract of producer strain Tue2. (A) Upper route (I), actinomycetes screening using the agar-based P_*clpE*_-lux bioreporter assay. Agar plugs from producer strain plates were embedded in P_*clpE*_-containing soft agar. Assessment of bioreporter induction (after 180 min) and zones of growth inhibition (after 18 h). Lower route (II), hit confirmation. Biomass was harvested from agar plates grown for 7 days and subjected to liquid–liquid partitioning with 1:1 (v/v) ethyl acetate (EtOAc) and water. Aqueous and organic extracts were evaluated for P_*clpE*_-lux induction, using filter discs for organic fractions and soft-agar wells for aqueous fractions. (B) Incubation of the representative μPAD section (spotted in a serpentine path) with the P_*clpE*_-lux bioreporter and the corresponding bioreporter activity chromatogram. Induction was detected in 5 of 500 spots within a short elution window (1.30–1.37 min), with maximal induction at 1.31 min. (C) The total ion chromatogram (TIC) shows a prominent peak at 1.31 min, which correlates with the maximal bioreporter response. (D) Mass spectrum at 1.31 min displaying adducts corresponding to streptothricin F (STP F), streptothricin E (STP E), and streptothricin D (STP D). (E) Streptothricin structural moieties, (I) carbamoylated gulosamine (black), (II) streptolidine lactam ring (red), and (III) variable length β-lysine homopolymer (green). (F) Annotated MS/MS fragmentation spectrum of STP F showing diagnostic ions from streptolidine cleavage (II) and β-lysine cleavage (III) relative to the gulosamine core (I). (G) Extracted ion chromatograms (XICs) for STP F (*m*/*z*: 503.2573 ± 10 ppm), STP E (*m*/*z*: 631.3522 ± 10 ppm), and STP D (*m*/*z*: 759.4472 ± 10 ppm), eluting at 1.31 min.

This approach facilitated the efficient isolation and characterization of highly polar bioactive metabolites that would otherwise be lost due to early elution in the dead volume of RP columns. Following incubation of the μPAD with the P_*clpE*_-lux bioreporter, luminescence signals from defined μPAD regions were mapped to their corresponding elution times to generate a bioreporter activity chromatogram (Figure 3B). Bioreporter induction was confined to a short elution window, with maximal induction observed at 1.31 min. Alignment of this bioreporter activity chromatogram with the LC-MS/MS total ion chromatogram (TIC) identified a prominent peak at 1.31 min (Figure 3C), consistent with the induction profile regarding retention time and peak shape. MS1 analysis at this retention time enabled precise identification of the candidate masses responsible for bioreporter induction within this short interval. The dominant feature was assigned to an [M + K]^+^ ion at *m*/*z* 541.2139 (calcd. 541.2137, +0.37 ppm), with the corresponding [M + H]^+^ ion at *m*/*z* 503.2580 (calcd. 503.2578, +0.40 ppm) (Figure 3D). Chemoinformatic analysis via SIRIUS^57^ (Figures S7 and S8) predicted these ions as streptothricin F (STP F). Two additional streptothricin analogs were identified by manual annotation, namely streptothricin E (STP E) and streptothricin D (STP D), each represented by multiple ion adducts.

Streptothricins inhibit protein synthesis by binding the 30S ribosomal subunit, blocking translocation, and inducing translational misreading.^58,59^ They are frequently encountered in bioactivity-guided natural product screens due to their potent activity and widespread occurrence in microbial extracts, resulting in substantial rediscovery burdens.^60–62^ Characteristic structural features include a carbamoylated gulosamine moiety (I), a streptolidine lactam ring (II), and a β-lysine homopolymer of variable length (III) (Figure 3E). Streptothricins naturally occur as mixtures of congeners, with individual members distinguished by the number of β-lysine residues, most commonly STP F (*n* = 1), STP E (*n* = 2), and STP D (*n* = 3). Manual MS/MS analysis, exemplified for STP F, confirmed the chemoinformatic prediction by identifying diagnostic ions corresponding to the individual structural components (Figure 3F). Cleavage of the β-lysine chain (III) generated fragment I + II, consisting of the carbamoylated gulosamine (I) and streptolidine lactam ring (II). Fragment I + III was generated by cleaving off the streptolidine lactam ring (II), resulting in the carbamoylated gulosamine (I) linked to the β-lysine residue (III). The combination of both cleavages produced the carbamoylated gulosamine core (I). Extracted ion chromatograms (XICs) confirmed STP F, STP E, and STP D as the bioactive compounds within the bioreporter induction window, coeluting at approximately 1.31 min (Figure 3G).

### Bioreporter-Guided Molecular Networking Reveals Streptothricin Diversity

Molecular networking was performed on the LC-MS/MS data set derived from the 53 aqueous extracts processed using the bioreporter-guided compound dereplication and discovery pipeline. A global molecular network was generated using the classical networking workflow in GNPS2 ^63^ (Figure 4A), enabling retention-time-based integration of bioreporter activity chromatograms for each bacterial isolate. Through this step, it was also possible to detect streptothricin masses in several extracts that did not elicit a bioreporter signal after microfractionation due to low compound abundance. The resulting molecular network revealed two distinct clusters of diverse STP derivatives, as supported by exact mass and diagnostic MS/MS fragmentation patterns (Figure 4B,4C). Within these clusters, a wide variety of STP congeners were dereplicated, ranging from STP F (one β-lysine residue) to STP B (five β-lysine residues), along with multiple derivatives bearing one to four putative acetylations, annotated based on MS/MS fragmentation patterns (level 3). STP resistance is classically attributed to monoacetylation of the β-amino group of the β-lysine moiety by STP acetyltransferases (Sats), which prevents ribosomal binding, representing a mechanism of producer self-resistance.^64^ Interestingly, we also detected the exact mass of a doubly acetylated STP F (calcd. 587.2784, found 587.2784, 0.00 ppm). The acetylation sites were confirmed by the distinct fragmentation pattern and by the creation of putative fragments (Figure S9). A similar observation was made for a tetra-acetylated STP D, which contains three β-lysine residues (calcd. 927.4894, found 927.4897, 0.32 ppm). The detection of several STP species with multiple acetylation modifications suggests a more complex modification pattern than previously described, including both multiple singly acetylated lysine residues per molecule and even doubly acetylated lysine residues.

**Figure 4:**
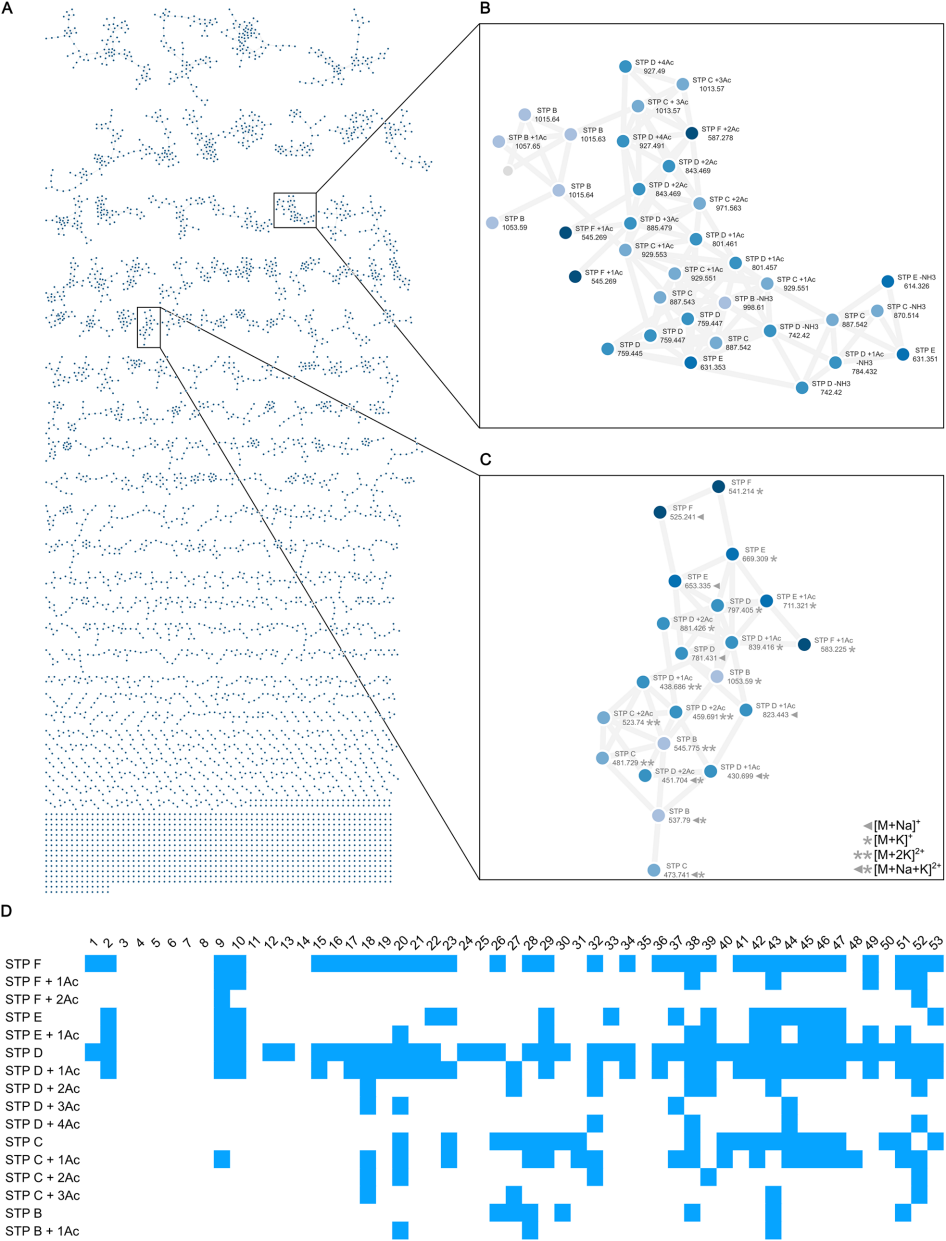
Identification of STP derivatives by molecular networking. (A) Global molecular network generated from LC-MS/MS data of the 53 aqueous extracts from isolates of the Tuebingen strain collection that induced the P_*clpE*_-lux bioreporter. Each node represents a unique MS/MS spectrum corresponding to a distinct molecular feature, and edges indicate spectral similarity based on calculated cosine scores. (B, C) Expanded views of two molecular clusters (B, [M + H]^+^; C, [M + K]^+^/[M + Na]^+^), corresponding to different STP congeners ranging from STP F to STP B (color-coded by congener class), including acetylation modifications; “+xAC” indicates the number of acetylation modifications detected. Edges reflect a high degree of spectral similarity between connected features. (D) A compound-presence matrix was constructed based on precursor masses and the fingerprint fragment (*m*/*z* = 171.0877), summarizing the distribution of identified STP derivatives (rows) across the producer strains (columns). In some cases, shifts in retention time occurred, which may indicate the presence of isobaric species. Blue squares indicate the presence of the corresponding molecule as determined by MS/MS analysis and the molecular network. STP identity is defined by β-lysine chain length: STP F (*n* = 1), STP E (*n* = 2), STP D (*n* = 3), STP C (*n* = 4), and STP B (*n* = 5).

To assess the distribution of STP derivatives across all bacterial isolates, a compound-presence matrix was constructed incorporating all dereplicated STP derivatives identified in the global network whose precursor masses and diagnostic fragment ions were consistent with STP structural features (Figure 4D). Among the 53 isolates from the Tuebingen strain collection that consistently induced the P_*clpE*_-lux bioreporter, STP derivatives were detected in 45 extracts, while 8 extracts lacked any identifiable STP congener. However, these eight extracts still contained diagnostic ions corresponding to the streptolidine lactam ring within the bioreporter induction window, suggesting the presence of previously uncharacterized STP variants. Ongoing work aims to identify these unknown STP derivatives and correlate metabolite distributions and bioactivity profiles with the phylogenetic taxonomy of the corresponding producer strains.

## Conclusions

This study establishes the P_*clpE*_-lux bioreporter as a sensitive diagnostic tool for the discovery of antibacterial compounds that induce proteotoxic stress through the accumulation of damaged or misfolded proteins. Covering this important mechanism shared by many natural products, including marketed antibiotics, broadens the detection capacity and MoA coverage of our bioreporter panel. The new P_*clpE*_-lux and our previously described P_*bmrC*_-lux^20^ bioreporters exhibit complementary activation profiles in response to ribosome-targeting antibiotics, differentiating between the generation of damaged proteins and translation arrest, respectively. The self-sustained luminescence enables continuous, real-time monitoring in both liquid and solid assay formats, supporting rapid screening without the need for extensive sample preparation. Our growing isogenic bioreporter panel in *B. subtilis* allows direct comparison of all induction signals, and the sporulation deficiency of the strain facilitates high-throughput handling. Validation of the P_*clpE*_-lux bioreporter performance with more than 430 natural products and antibacterial reference compounds identified several agents whose MoA had not previously been associated with proteotoxic stress. For well-characterized agents, our results hint at putative secondary or downstream effects of primary MoAs and potential mechanistic differences among representatives of the same antibiotic classes. For less investigated compounds, MoA hypotheses emerge. Follow-up investigations, such as transcriptomic profiling or miscoding assays, will be required to confirm these effects and elucidate the underlying molecular mechanisms. In aqueous extracts of producer strains from the Tuebingen actinomycete collection, P_*clpE*_-lux-guided analysis of microfractions substantially accelerated the dereplication of hydrophilic bioactive agents. The combination of bioreporter activity profiles with high-resolution MS/MS data in the same dereplication pipeline reduced the data complexity and enabled efficient identification of compounds of interest by aligning retention times with bioactivity peak shapes. This integrative approach allowed the accurate annotation of STP congeners across the producer strain collection and suggests previously unnoted structural diversity. Spectral similarity clustering facilitated the recognition of potentially uncharacterized STP variants and derivative families. Moreover, as STPs are widespread natural products, the comprehensive annotation of their derivative space will improve future de novo dereplication workflows. Collectively, this work presents a flexible platform for the rapid discovery and dereplication of urgently needed novel antibiotics, building on the sensitivity, specificity, and broad applicability of the bioreporter technology. Combining bioreporter-guided microfractionation with molecular networking provides a versatile strategy for identifying bioactive compounds and elucidating their structural relationships. This adaptable pipeline can be readily applied across diverse bioassay formats and screening methodologies to accelerate antibiotic discovery.

## Experimental Section

### General Experimental Procedures

Standard molecular cloning procedures, including restriction–ligation and Gibson assembly, were performed using established protocols. Plasmid DNA was isolated using the GeneJET Plasmid Miniprep Kit (Thermo Scientific) according to the manufacturer’s instructions. DNA sequencing was performed using Sanger sequencing (LGC Genomics GmbH). Luminescence imaging in the agar-based bioreporter assay was conducted using a ChemiDoc MP imaging system (Bio-Rad). In the liquid-based bioreporter assay, luminescence and optical density (OD_600_) were measured using a SPARK multimode microplate reader (Tecan). Microfractionation was conducted using a custom-made high-speed microfractionation device (Microspotter) combined with a microfluidic paper-based analytical device (μPAD), as reported previously.^20^ Chromatographic separation was performed on an Agilent 1260 Infinity II system (Agilent Technologies) equipped with a quaternary pump, autosampler, and UV/vis detector. Mass spectrometric detection was performed on a Q Exactive Orbitrap (Thermo Fisher Scientific).

### Bioreporter Generation and Cultivation

The P_*clpE*_-lux bioreporter was constructed by transforming the sporulation-deficient *B. subtilis* strain 1S34 ^31^ with a modified version of the integrative shuttle reporter vector pBS3Clux.^45^ The *clpE* promoter region (P_*clpE*_) was amplified from *B. subtilis* 1S34 genomic DNA and cloned into pBS3Clux, positioning P_*clpE*_ 19 bp upstream of the *P. luminescens* *luxABCDE* operon. The vector was propagated in *Escherichia coli* XL-10, cultured in Lysogeny Broth (LB) with 100 μg/mL ampicillin, followed by plasmid isolation and verification of correct promoter insertion via sequencing. Naturally competent *B. subtilis* 1S34 cells were prepared by nutrient starvation^65^ and transformed with the assembled construct, which integrated into the chromosomal *sacA* locus. Transformants were selected on LB agar containing 5 μg/mL chloramphenicol (CAM), and genomic integration was verified by colony polymerase chain reaction (PCR). Primers used in this study are listed in Table S2. The P_*clpE*_-lux bioreporter was routinely cultured in LB with 5 μg/mL CAM at 37 °C and agitation at 190 rpm.

### Agar-Based Bioreporter Assay

A glycerol stock of the P_*clpE*_-lux bioreporter was used to inoculate a preculture (18 h), which was subsequently diluted to an OD_600_ of 0.05, and the main culture was grown to an OD_600_ of ≈1.0. LB soft agar (0.75% agar, without CAM) was inoculated with the main culture to a final cell count of 3 × 10^6^ CFU/mL, poured into 120 × 120 mm square plates (Sarstedt), and solidified for 20 min. Pure compounds (≤5 μL) were applied directly onto the agar surface, whereas larger sample volumes were applied using sterile filter discs or wells punched into the agar using a cork borer. For natural producer strains, prestamped agar blocks (≥7 days of cultivation) were placed in the plate and embedded in soft agar inoculated with the bioreporter. For μPAD assays, the bioreporter soft agar was poured onto μPAD sheets placed on a thin underlayer of sterile soft agar. Plates were incubated at 37 °C for 180 min, and luminescence (λ = 490 nm) was recorded in a ChemiDoc imager equipped with a cooled charge-coupled device (CCD) detector in chemiluminescent blot 647SP mode with an exposure time of 600 s. Luminescence images were processed using Fiji.^66^ Contrast was enhanced for each image using the “Enhance Contrast” function (0.2% saturated pixels, normalization enabled). To ensure consistent visualization of luminescence signals across the DZIF screening plates, a defined background region was selected on a randomly chosen assay plate to measure the baseline luminescence of the P_*clpE*_-lux bioreporter. The remaining plates were then adjusted so that their background intensities matched that of the reference plate. Intrinsic CCD detector noise, including readout noise and dark current, becomes more prominent at low signal levels, making assay plates with low overall luminescence appear noisier. After imaging, plates were incubated for 18 h at 30 °C to assess antibacterial activity by measuring zones of inhibition.

### Liquid-Based Bioreporter Assay

Precultures and main cultures were prepared as described for the agar assay. Reference antibiotics were serially diluted 2-fold in prewarmed LB using white, clear, flat-bottom 96-well plates (Brand). The P_*clpE*_-lux bioreporter was diluted in prewarmed LB medium (without CAM), and 60 μL were added to compound-containing wells to yield a final cell count of 1 × 10^7^ CFU/mL in 120 μL total volume. Luminescence and optical density were recorded in a multimode microplate reader at 5 min intervals over 180 min under incubation conditions specified in Table S3. Bioreporter induction was defined as a ≥500% increase in luminescence within 90 min of antibiotic exposure relative to the baseline signal of the untreated control (100%). This threshold was conservatively defined based on the distribution of induction responses across the panel of reference compounds used for validation, ensuring that all known proteotoxic stress inducers exceeded the cutoff, whereas compounds with unrelated mechanisms consistently remained below it.

### Natural Producer Strain Cultivation and Extraction

Natural producer strains from the Tuebingen strain collection were cultivated on ISP2 and ISP3 agar in 120 × 120 mm square plates (Sarstedt) for 7 days at 28 °C. Biomass was harvested using a cell scraper (Sarstedt) and transferred into an equal volume of water/ethyl acetate (1:1). Extraction was performed in an overhead shaker for 18 h, followed by phase separation via centrifugation (4000*g*, 10 min, 4 °C). The aqueous and organic layers were collected separately and lyophilized. Dried organic extracts were reconstituted in 80% methanol, and aqueous extracts were reconstituted in deionized water.

### Microfractionation, LC-MS/MS Analysis, and Molecular Networking

Instrumentation for microfractionation, chromatographic separation, and mass spectrometric detection is described under General Experimental Procedures. Due to the strong polarity of the bioactive metabolites, samples were separated on a Hypercarb column (100 × 4.6 mm, 5 μm; Thermo Fisher Scientific). Mobile phases consisted of LC-MS-grade water (A) and acetonitrile (B), both containing 0.1% formic acid. The gradient profile was 0.00–2.00 min, 2% B; 2.00–12.00 min, linear increase to 99% B; 12.00–13.00 min, hold at 99% B; 13.01 min, return to 2% B; total runtime 15 min; flow rate 1 mL/min. Full MS scans were acquired in positive ion mode from *m*/*z* 150–2000 at a resolution of 35,000. Source parameters were sheath gas 35 AU, auxiliary gas 5 AU, sweep gas off, spray voltage 3.5 kV, capillary temperature 250 °C, and auxiliary gas heater temperature 250 °C. Molecular networking was conducted using the classical networking workflow in GNPS2 ^63^ with the following parameters: precursor and fragment ion tolerance 0.002 Da; minimum cluster size 2; minimum cosine score (library and network) 0.7; and minimum matched peaks 6. Networks were visualized using Cytoscape. In silico annotation and molecular formula calculations of MS/MS data were performed using SIRIUS 6.^57^

## Supplementary Material





## Data Availability

LC-MS/MS raw and processed files are available through the Zenodo repository: https://zenodo.org/records/18186652 (raw) and https://zenodo.org/records/18198130 (mzML). GNPS2 molecular networking results are available through https://gnps2.org/status?task=f25d1c6bfc3e44fdaebe12d81124b24e.

## Notes

Supporting Information File 1: DZIF library compounds (XLSX)
Media composition (Table S1), primer sequences (Table S2), microplate reader settings (Table S3), antibacterial reference compounds (Table S4), bioreporter assay controls (Figures S1–S6), SIRIUS predictions (Figures S7, S8), and STP F + 2Ac prediction (Figure S9) (PDF)
