# Supplementary material for: Proteotoxic Stress Bioreporter Enables Mechanism-Informed Antibiotic Discovery
Source: J Nat Prod. 2026 May 15;89(6):1698–710. doi: 10.1021/acs.jnatprod.6c00112 (PMC13316988; doi:10.1021/acs.jnatprod.6c00112)
Supplement: Supplementary file 2 [file np6c00112_si_002.pdf]

Supplementary Information

A Proteotoxic Stress Bioreporter Enables Mechanism-Informed Antibiotic Discovery

Julian Schubert[1,2], Christian Geibel[1,2], Anne Berscheid[1,3], Katharina W. Wex[1,3], Giovanni Andrea Vitale[1,2], Chambers C. Hughes[1,2,3], Daniel Petras[2,4,*], Heike Brötz-Oesterhelt[1,2,3,*]

1. Department of Microbial Bioactive Compounds, Interfaculty Institute of Microbiology and Infection Medicine (IMIT), University of Tuebingen, 72076 Tuebingen, Germany
2. Cluster of Excellence EXC 2124: Controlling Microbes to Fight Infection (CMFI), University of Tuebingen, 72076 Tuebingen, Germany
3. German Center for Infection Research (DZIF), Partner Site Tuebingen, 72076 Tuebingen, Germany
4. Department of Biochemistry, University of California Riverside, Riverside, California 92507, USA

*Corresponding authors: dpetras@ucr.edu; heike.broetz-oesterhelt@uni-tuebingen.de

Table of Contents

Table S1. Media composition. 3
Table S2. Primers used in this study. 3
Table S3. Tecan Spark multimode microplate reader settings. 4
Table S4. Antibacterial reference compounds used in the validation of the P_{clpE}-lux bioreporter 5
Fig. S1. P_{clpE}-lux bioreporter validation using established proteotoxic stress inducers 8
Fig. S2. P_{clpE}-lux bioreporter validation using protein synthesis inhibitors. 9
Fig. S3. P_{clpE}-lux bioreporter validation using cell envelope inhibitors and fatty acid synthesis inhibitors 10
Fig. S4. P_{clpE}-lux bioreporter validation using DNA and RNA synthesis inhibitors, intercalators, and antibacterial agents with diverse MoAs 11
Fig. S5. Representative agar validation plates 13
Fig. S6. Exemplary screening plates from the DZIF library 14
Fig. S7. SIRIUS 6 ranking of candidate molecular formulas for the ion at *m/z* 503.258015
Fig. S8. SIRIUS 6 ranking of candidate structures for the ion at *m/z* 503.2580 15
Fig. S9. STP F +2Ac prediction. 16
References 16

Table S1. Media composition.

Medium	Composition
LB	1% NaCl, 1% tryptone, 0.5% yeast extract, pH 7.25
LB soft agar	1% NaCl, 1% tryptone, 0.5% yeast extract, 0.75% agar, pH 7.25
ISP2	0.4% D-glucose, 1% malt extract, 0.4% yeast extract, 2% agar, pH 7.3
ISP3	2% oatmeal, 0.5% trace elements, 1.5% agar, pH 7.3
Trace-elements	0.3% $CaCl_2 \cdot 2H_2O$, 0.1% Fe(III)-citrate, 0.02% $MnSO_4 \cdot 1H_2O$, 0.01% $ZnCl_2$, 0.0025% $CuSO_4 \cdot 5H_2O$, 0.002% $Na_2B_4O_7 \cdot 10H_2O$, 0.0004% $CoCl_2 \cdot 6H_2O$, 0.001% $Na_2MoO_4 \cdot 2H_2O$

Table S2. Primers used in this study.

PCR	Nucleotide sequence (5'-3')
ClpE-fwd	GATAAGCTGTCAAACATGAGAATTCATGTTTTACTTTGTAACAAATCAA
ClpE-rev	TTTCATAGAGAGTCCTCCTGTCGACGGAATTTATTTGCATGTTAAGGC
**Sequencing**	**Nucleotide sequence (5'-3')**
Insert-fwd	TTCGTTTGTTGAACTAATGGGTGC
Insert-rev	AAACCACACTCCTCAGAGATG
**Colony PCR**	**Nucleotide sequence (5'-3')**
SacA-fwd	CTGATTGGCATGGCGATTGC
SacA-rev	ACAGCTCCAGATCCTCTACG

Table S3. Tecan Spark multimode microplate reader settings.

Parameter	Settings
Lid	Yes
Humidity cassette	No
Temperature control	On, wait for temperature
Temperature	37°C [min: 36°C, max: 38°C]
Measurement	180 min
Interval	5 min
Shaking	60 sec, double orbital, amplitude 2.5 mm (108 rpm)
Wait	10 sec
Luminescence	Integration time 1000 ms
Absorbance	Wavelength 600 nm, bandwidth 3.5 nm, flashes 10, settle time 100 ms, multiple reads per well: XY-line 3 x 3, border 2400 µm
Wait	Wait for interval restart

Table S4. Antibacterial reference compounds used in the validation of the P_{clpE}-lux bioreporter. Compounds eliciting bioreporter induction are denoted by "+", whereas non-inducing compounds are marked with "−".

Compound	Agar	Liquid
**Proteotoxic stress inducers and protein synthesis inhibitors**		
Abortive translation inducer		
Puromycin	+	+
Misreading inducers		
Amikacin	+	+
Apramycin	+	+
Gentamicin	+	+
Hygromycin B	+	+
Kanamycin	+	+
Neomycin	+	+
Paromomycin	+	+
Streptomycin	+	+
Tobramycin	+	+
Thiol-modifying agents		
N-ethylmaleimide	+**	+
TMAD	+	+
Clp protease dysregulators		
ADEP1	+	+
ADEP2	+	+
ADEP7	+	+
Translation stallers		
Anhydrotetracycline	-	-
Avilamycin B*	-	+
Avilamycin C*	-	+
Azithromycin	-	-
Berninamycin C*	+	+
Chloramphenicol	-	-
Clindamycin	-	-
Doxycycline	-	-
Erythromycin	-	-
Fusidic acid*	+	+
Kirromycin	-	-
Kirrothrycin	-	-
Lincomycin	-	-
Linezolid	-	-
Oxamicetin	-	-
Pactamycin	-	-
Spectinomycin	-	-
Sulfomycin I	-	-
Telithromycin	-	-
Tetracycline	-	-

Compound	Agar	Liquid
Thiostrepton	-	-
Tiamulin	-	-
Tigecycline	-	-
tRNA synthetase inhibitors		
Mupirocin	-	-
**Cell envelope inhibitors**		
Cell membrane disruptors		
Colistin	-	-
Mefloquine	-	-
Polymyxin B	-	-
Ionophores		
CCCP	-	-
Monensin	-	-
Nigericin	-	-
Salinomycin	-	-
Lipid-II cycle inhibitors		
A-47934	-	-
Bacitracin	-	-
Daptomycin	-	-
Mersacidin	-	-
Nisin	-	-
Ramoplanin	-	-
Teicoplanin	-	-
Vancomycin	-	-
Peptidoglycan synthesis enzyme inhibitors		
Ampicillin	-	-
Benzylpenicillin	-	-
Cefadroxil	-	-
Cefalexin	-	-
Cefotaxime	-	-
Cefoxitin	-	-
Cefuroxime	-	-
D-Cycloserine	-	-
Fosfomycin	-	-
Imipenem	-	-
Meropenem	-	-
Methicillin	-	-
Oxacillin	-	-
**Fatty acid synthesis inhibitors**		
Triclosan	-	-
**DNA synthesis inhibitors**		
DNA gyrase binders		
Ciprofloxacin	-	-
Levofloxacin	-	-
Moxifloxacin	-	-

Compound	Agar	Liquid
Nalidixic acid	-	-
Norfloxacin	-	-
Novobiocin	-	-
Nucleotide synthesis inhibitors		
Azaserine	-	-
Trimethoprim	-	-
**RNA synthesis inhibitors**		
RNA polymerase binders		
Fidaxomicin	-	-
Rifabutin	-	-
Rifampicin	-	-
**Intercalators**		
Actinomycin D	-	-
Cisplatin	-	-
Doxorubicin	-	-
Echinomycin	-	-
Emodin	-	-
Mitomycin C	-	-
Mitoxantrone	-	-
Netropsin	-	-
Proflavine	-	-
Zeocin	-	-
**Diverse mechanisms of action**		
5'-Fluorouracil	-	-
Holomycin	-	-
Nitrofurantoin	-	-
Thiolutin	-	-
Derinamycin	-	-
Rinamycin	-	-
Spermidine	-	-
Spermine	-	-
Frigocyclinone	-	-
Quinine	-	-

* Categorized as “translation stallers” per published mechanism of action (MoA); P_{clpE}-lux induction in the current study suggests additional accumulation of damaged or misfolded proteins, a hypothesis requiring assessment by complementary methods.
** Transient induction of P_{clpE}-lux at 90 min after antibiotic exposure.

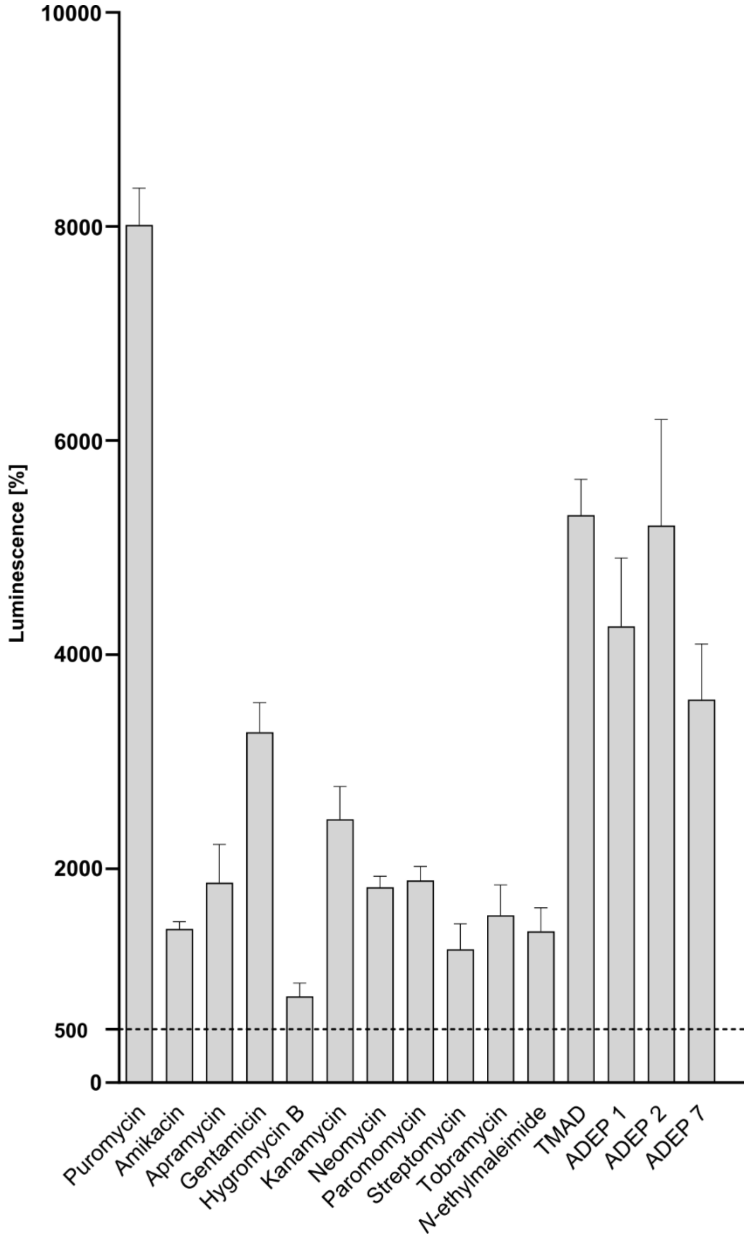

Fig. S1. P_{clpE}-lux bioreporter validation using established proteotoxic stress inducers. Quantitative bioreporter induction by reference compounds in the liquid assay format. The compounds correspond to those summarized in Table S4 and include abortive translation inducers, misreading inducers, thiol-modifying agents, and Clp protease dysregulators. Compounds were tested in two-fold serial dilutions, from below to above their minimal inhibitory concentrations (MIC). Luminescence and OD_{600} (cell mass) were monitored for 180 min, and induction was normalized to the baseline luminescence of the untreated control (100%) and the OD_{600}. The induction threshold was defined as ≥500% luminescence within 90 min of antibiotic exposure (black dotted line). Bars represent maximal normalized luminescence (mean ± SD) from independent biological replicates.

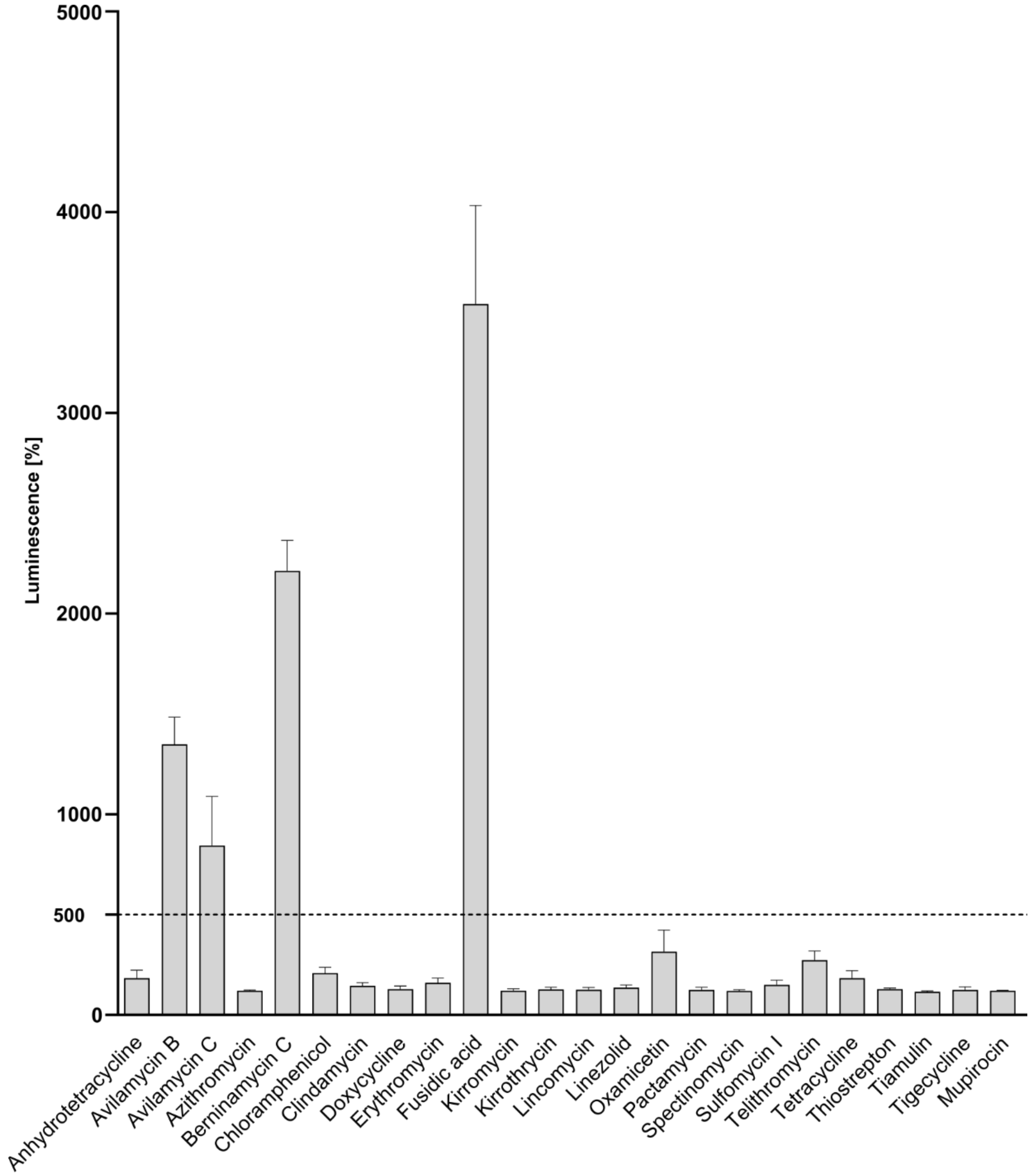

Fig. S2. P$_{clpE}$-lux bioreporter validation using protein synthesis inhibitors. Quantitative bioreporter induction by reference compounds in the liquid assay format. The compounds correspond to those summarized in Table S4 and include translation stallers and tRNA synthetase inhibitors. Compounds were tested in two-fold serial dilutions, from below to above their minimal inhibitory concentrations (MIC). Luminescence and OD_{600} (cell mass) were monitored for 180 min, and induction was normalized to the baseline luminescence of the untreated control (100%) and the OD_{600}. The induction threshold was defined as ≥500% luminescence within 90 min of antibiotic exposure (black dotted line). Bars represent maximal normalized luminescence (mean ± SD) from independent biological replicates.

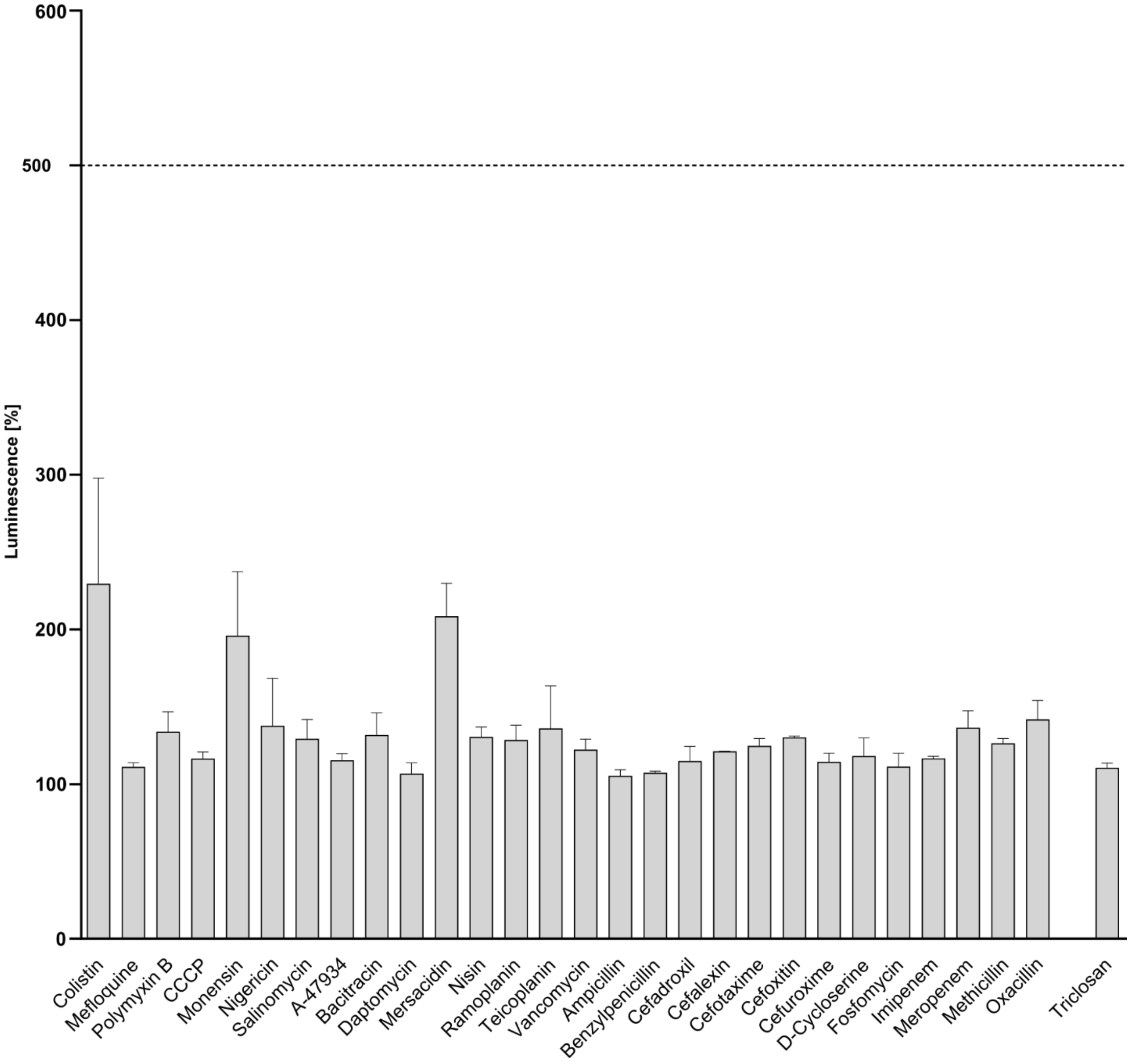

Fig. S3. P_{clpE}-lux bioreporter validation using cell envelope inhibitors and fatty acid synthesis inhibitors. Quantitative bioreporter induction by reference compounds in the liquid assay format. The compounds correspond to those summarized in Table S4 and include cell membrane disruptors, ionophores, lipid-II cycle inhibitors, peptidoglycan synthesis enzyme inhibitors, and the fatty acid synthesis inhibitor triclosan. Compounds were tested in two-fold serial dilutions, from below to above their minimal inhibitory concentrations (MIC). Luminescence and OD_{600} (cell mass) were monitored for 180 min, and induction was normalized to the baseline luminescence of the untreated control (100%) and the OD_{600}. The induction threshold was defined as ≥500% luminescence within 90 min of antibiotic exposure (black dotted line). Bars represent maximal normalized luminescence (mean ± SD) from independent biological replicates.

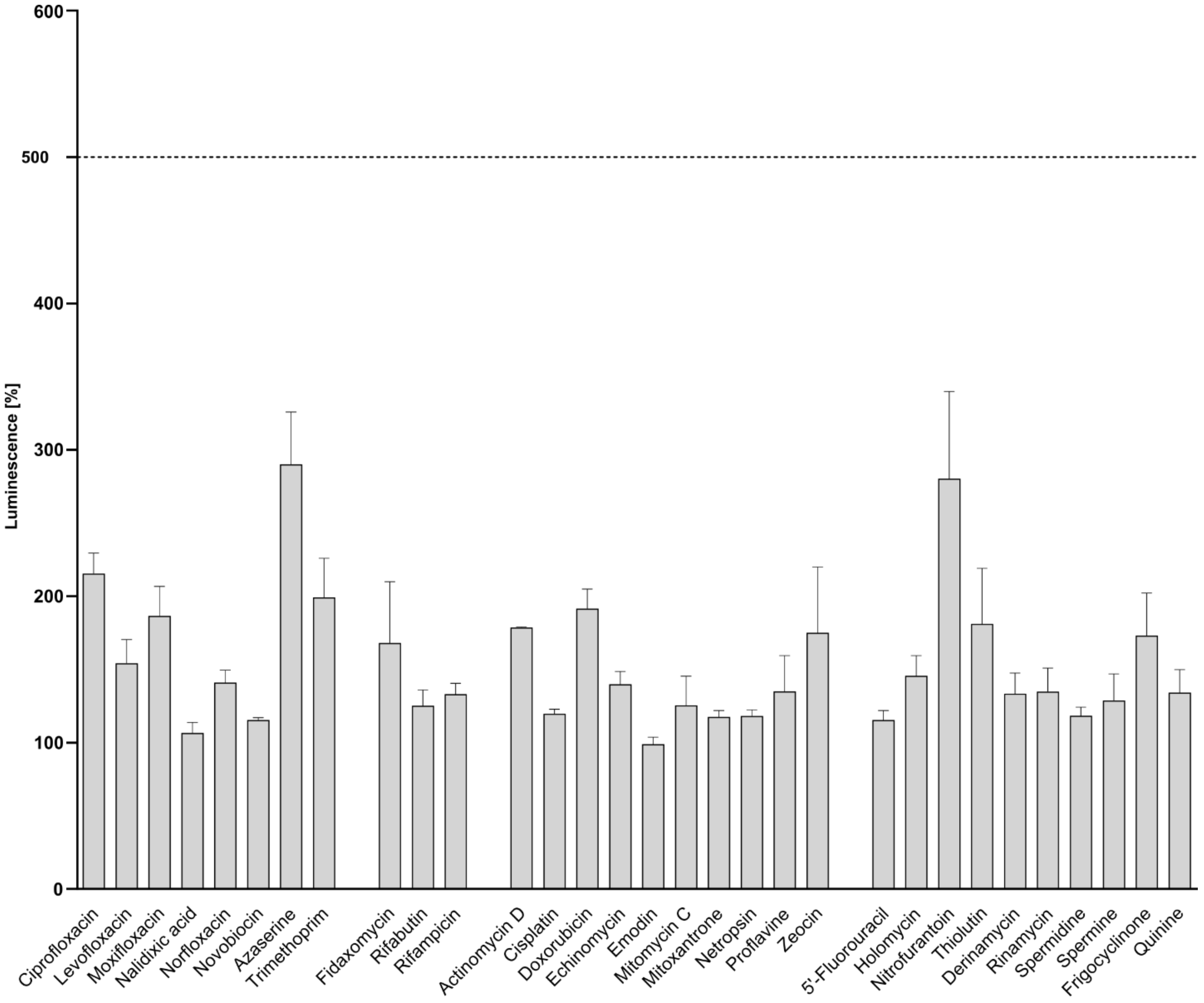

Fig. S4. P_{clpE}-lux bioreporter validation using DNA and RNA synthesis inhibitors, intercalators, and antibacterial agents with diverse MoAs. Quantitative bioreporter induction by reference compounds in the liquid assay format. The compounds correspond to those summarized in Table S4 and include DNA gyrase binders, nucleotide synthesis inhibitors, RNA polymerase binders, intercalators, and compounds with diverse MoAs. Compounds were tested in two-fold serial dilutions, from below to above their minimal inhibitory concentrations (MIC). Luminescence and OD_{600} (cell mass) were monitored for 180 min, and induction was normalized to the baseline luminescence of the untreated control (100%) and the OD_{600}. The induction threshold was defined as ≥500% luminescence within 90 min of antibiotic exposure (black dotted line). Bars represent maximal normalized luminescence (mean ± SD) from independent biological replicates.

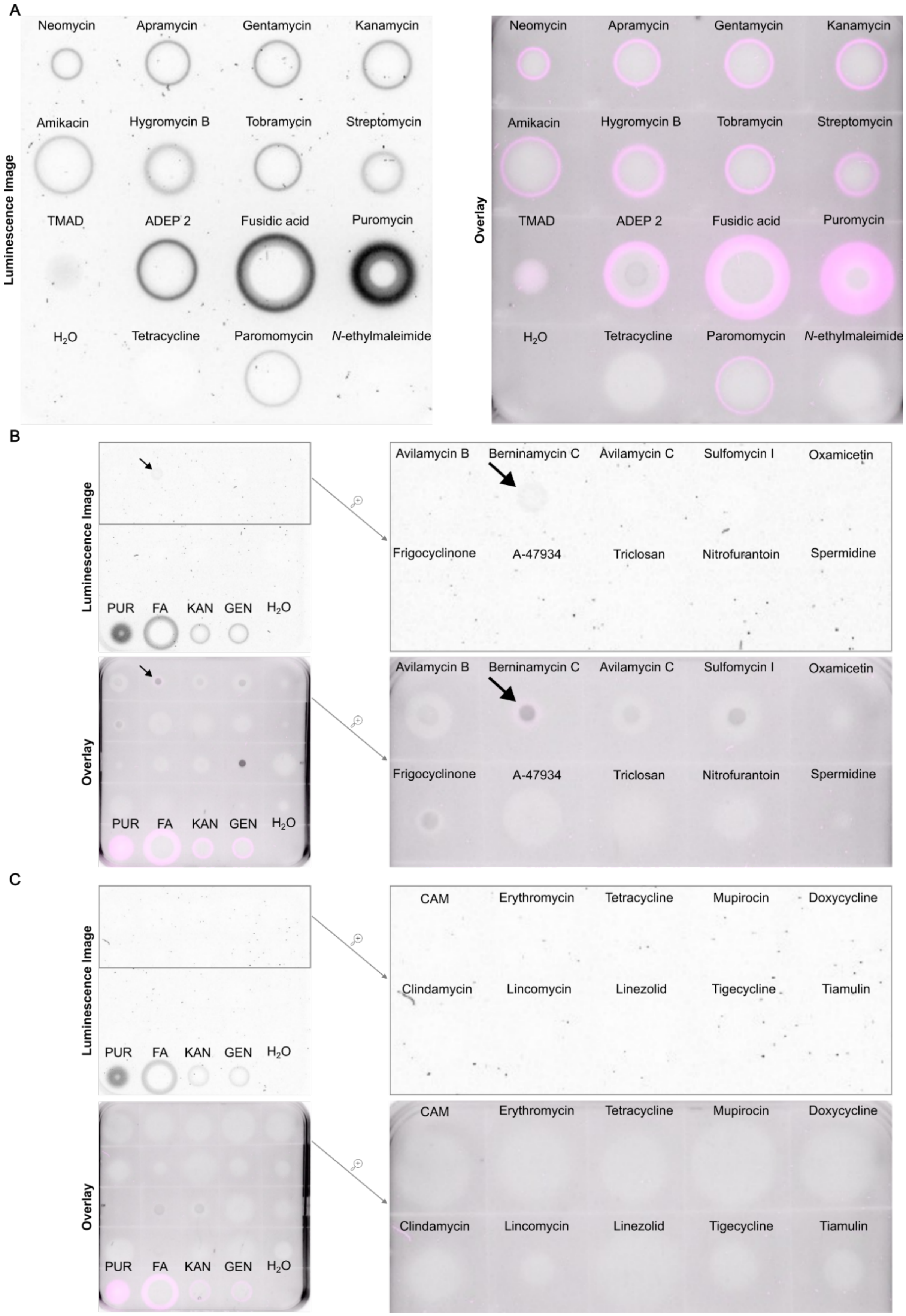
A
Luminescence Image
Neomycin
Apramycin
Gentamycin
Kanamycin
Amikacin
Hygromycin B
Tobramycin
Streptomycin
TMAD
ADEP 2
Fusidic acid
Puromycin
H_2O
Tetracycline
Paromomycin
N-ethylmaleimide
Overlay
B
PUR
FA
KAN
GEN
Avilamycin B
Berninamycin C
Avilamycin C
Sulfomycin I
Oxamicetin
Frigocyclinone
A-47934
Triclosan
Nitrofurantoin
Spermidine
C
CAM
Erythromycin
Mupirocin
Doxycycline
Clindamycin
Lincomycin
Linezolid
Tigecycline
Tiamulin

Fig. S5. Representative agar validation plates. Validation of the P_{clpE}-lux bioreporter using selected reference compounds in the agar-based assay format. Compounds were spotted either directly onto the bioreporter lawn or applied on filter discs in a 4 × 4 (A) or 5 × 5 (B, C) grid and imaged after 180 min. For each agar plate, both the luminescence image (labeled "luminescence image") and a corresponding overlay image (labeled "overlay"), in which the luminescence image is superimposed in pink onto the epi-luminescence image showing growth inhibition zones, are presented. Selected reference compounds include established proteotoxic stress inducers, non-inducing compounds, and the unexpected inducers fusidic acid and berninamycin C, as well as water (H_2O) as a negative control. The same set of reference compounds was applied to each plate, including puromycin (PUR), fusidic acid (FA), kanamycin (KAN), and gentamicin (GEN). In panels (B) and (C), regions of the agar plates are enlarged to better visualize the weak induction observed for berninamycin C, the absence of induction for avilamycin B and C in the agar-based set-up (while the avilamycin B and C both induced in the liquid setup, Fig. 1 and Fig. S2), and the lack of detectable luminescence on agar for non-inducing reference compounds despite visible zones of growth inhibition.

Based on our prior experience with various bioreporter strains, most inducing agents produce detectable signals in liquid and solid media, but we also encountered exceptional agents that produce signals only in one of these setups.[1] This observation was not specific to the P_{clpE}-lux bioreporter but applied to other bioreporters as well. Such differences may reflect variations in bacterial physiology under these distinct growth conditions, resulting in differential sensitivity to certain compounds or divergent coping mechanisms of the bioreporter strain in these setups.

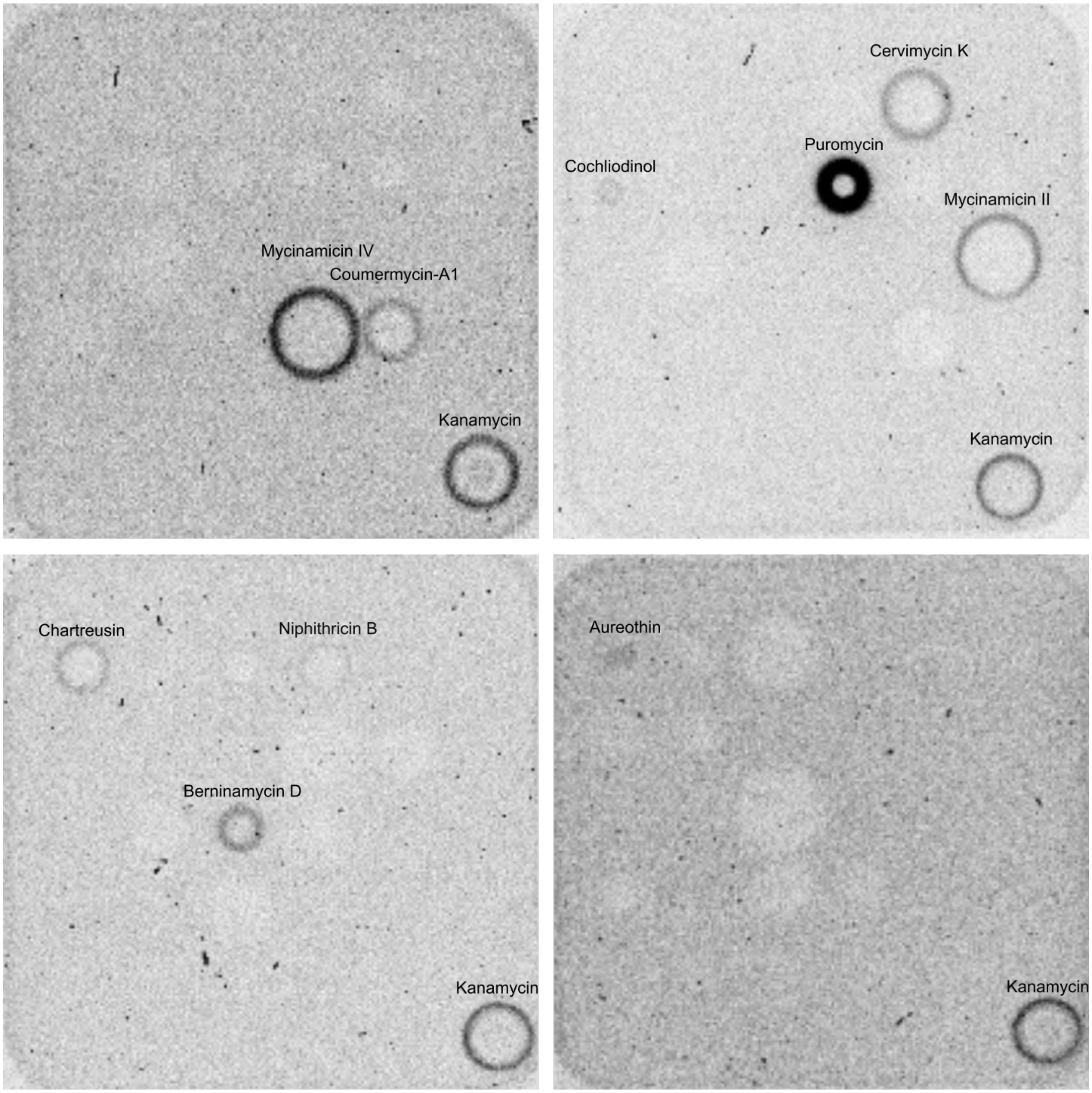

Fig. S6. Exemplary screening plates from the DZIF library. The compounds were directly spotted onto the bioreporter lawn in a 5 × 4 grid, with kanamycin included as a positive control in the bottom-right corner of each plate. Full, uncropped luminescence images of four representative screening plates are shown, acquired after 180 min. Displaying the complete plates allows direct comparison of inducing and non-inducing compounds. Natural variations in background noise arise from the intrinsic properties of the CCD detector (including readout noise and dark current), which become more prominent at low overall signal levels.

Rank	Molecular Formula	Adduct	Zodiac Score	Sirius Score (normalized)	Isotope Score	Tree Score	Explained Peaks	Total Explained Intensity	Median Mass Error (ppm)	Median Mass Error (mDa)
1	C19H34N8O8	[M + H]+	NaN	99,237%	6,219	89,139	49/49	97,955%	1,314	0,255
2	C18H38N4O12	[M + H]+	NaN	0,696%	5,312	85,085	48/49	97,738%	3,461	0,509
3	C24H34N6O6	[M + H]+	NaN	0,067%	2,728	85,332	49/49	97,955%	1,032	0,174
4	C23H38N2O10	[M + H]+	NaN	0,000%	4,371	77,713	47/49	97,463%	-1,400	-0,306
5	C27H35FN2O6	[M + H]+	NaN	0,000%	1,655	63,760	42/49	91,839%	0,727	0,144
6	C16H35FN8O9	[M + H]+	NaN	0,000%	5,878	43,926	32/49	90,650%	1,408	0,259
7	C17H39N6O9P	[M + H]+	NaN	0,000%	5,681	43,443	32/49	90,650%	1,408	0,259
8	C15H30N14O6	[M + H]+	NaN	0,000%	4,272	43,559	32/49	90,650%	2,661	0,370
9	C17H33F3N8O6	[M + H]+	NaN	0,000%	4,882	42,463	32/49	90,650%	2,661	0,370
10	C20H30N12O4	[M + H]+	NaN	0,000%	5,751	40,048	30/49	89,150%	1,489	0,250

Fig. S7. SIRIUS 6 ranking of candidate molecular formulas for the ion at *m/z* 503.2580. C19H34N8O8 is ranked as the most probable formula.

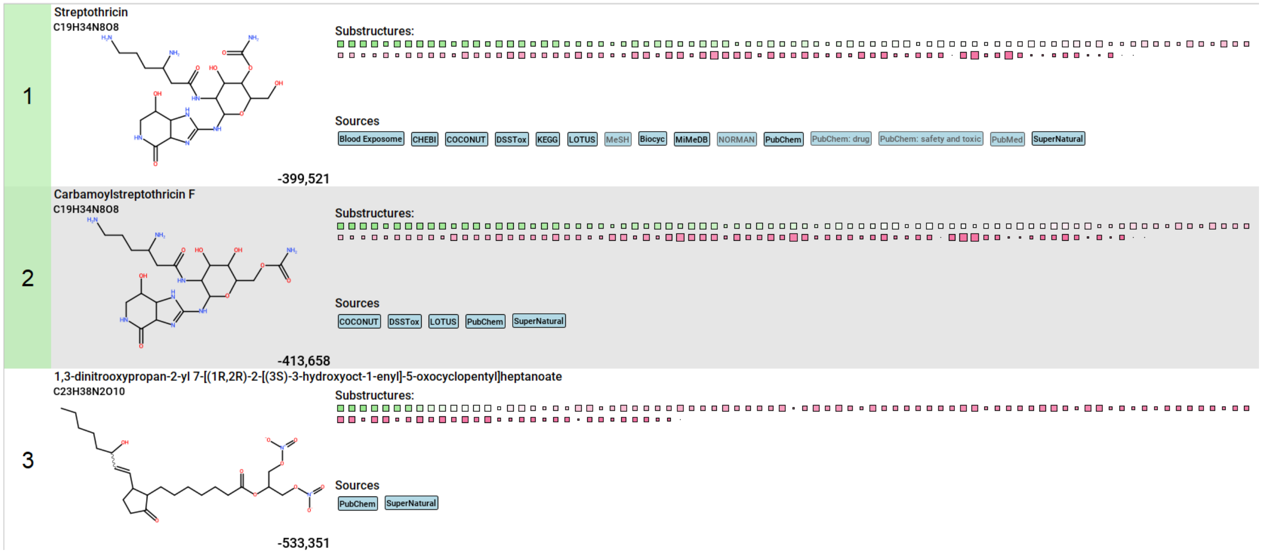

Fig. S8. SIRIUS 6 ranking of candidate structures for the ion at *m/z* 503.2580. STP F is ranked as the most probable structure.

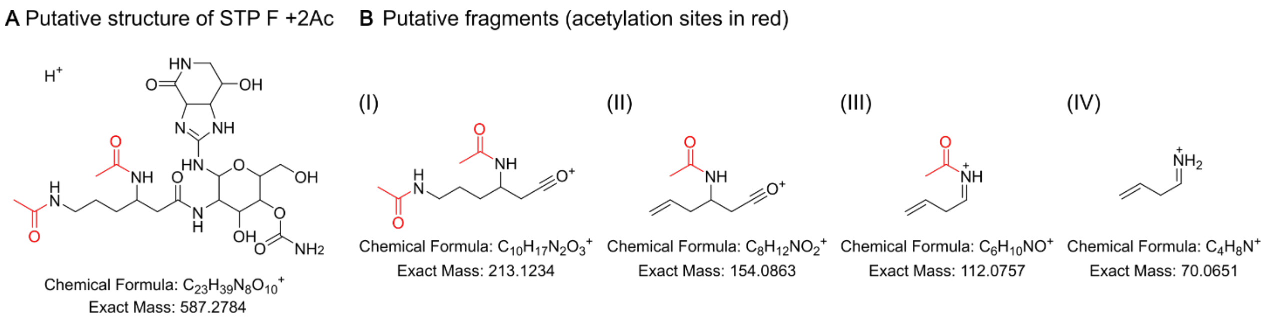

C

Calcd. Mass [Da]	Found Fragments [*m/z*]	Mass Error [ppm/mDa]	Explanation
587.2784	587.2755	-4.94/2.9	Parent mass
213.1234	213.1234	0.00/0	Putative fragment (I)
171.0877	171.0877	0.00/0	STP reporter ion
154.0863	154.0863	0.00/0	Putative fragment (II)
112.0757	112.0760	2.68/0.3	Putative fragment (III)
70.0651	70.0658	9.99/0.7	Putative fragment (IV)

Fig. S9. STP F +2Ac prediction. A) Putative structure of STP F with two acetylation sites on both primary amines of the β-lysine moiety. The mass was confirmed on MS1 level with a mass error of 0.00 ppm. B) Putative fragments for the doubly acetylated β-lysine moiety. Acetylation sites are highlighted in red. C) Overview of the calculated and found *m/z* for the MS2 of 587.2784. Apart from the reporter ion for streptothricins (171.0877), four distinct fragment ions matching the predictions were found.

References

(1) Geibel, C.; Schubert, J.; Knoblauch, S. B.; Hernandez, A.; Boldt, L.; Schneider, D. C.; Papadopoulos Lambidis, S.; Vitale, G. A.; Fleischer, J.; Haussmann, M.; Gross, H.; Wang, M.; Brötz-Oesterhelt, H.; Petras, D. High-Frequency Microfluidic Fractionation for Compound-Resolved Bioactivity-Based Metabolomics. *Anal. Chem.* **2025**, *97* (43), 24093–24104. https://doi.org/10.1021/acs.analchem.5c04612.
